# A Preliminary Study of the Attitudes and Barriers of Family Physicians to Prescribing HIV Preexposure Prophylaxis

**Published:** 2017-05-15

**Authors:** Nicholas Ojile, Donna Sweet, K. James Kallail

**Affiliations:** Department of Internal Medicine, University of Kansas School of Medicine-Wichita, KS

## Abstract

**Introduction:**

Attitudes of individuals who provide HIV care towards prescribing Preexposure Prophylaxis (PrEP) to at-risk populations have been studied, but few studies indicate if family physicians would be willing to prescribe PrEP as most family physicians do not specialize in HIV medicine. Few data exist on the perceived barriers preventing family physicians from prescribing PrEP. The purpose of this project was to assess the attitudes and perceived barriers of family physicians in Kansas towards prescribing PrEP to high risk patient populations.

**Methods:**

This study was a descriptive, observational, and cross-sectional survey of family physicians who respond to email surveys issued through the Family Medicine Research and Data Information Office (FM RADIO).

**Results:**

Fifty-three percent of family physicians take a sexual history on new patients less than frequently, and only 35% frequently ask about the use of safe sex practices. Only 29% frequently ask if the patient has sex with men, women, or both. Seventy-six percent of respondents would be willing to prescribe PrEP to men who have sex with men, and an equal percentage would be willing to prescribe to heterosexually active men and women who are at substantial risk of acquiring HIV. While 59% of participants agreed that PrEP belongs in the primary care domain of treatment, 71% agreed that they had limited or no knowledge of PrEP guidelines.

**Conclusions:**

This preliminary study indicated a need for increased family physician screening of new patients for high risk sexual behaviors who would be eligible for PrEP. The limited knowledge of PrEP guidelines and its use in clinical practice are significant limiting factors to increasing prescribing practices in the family medicine community rather than a perceived ethical dilemma of prescribing PrEP to men who have sex with men. As a result, an increase in continuing medical education about PrEP could significantly increase its prescribing in the family medicine community.

## Introduction

Over 1.2 million people live with HIV in the United States, with 50,000 new infections diagnosed each year.^1^,^2^ The US Centers for Disease Control and Prevention (CDC) projects the lifetime risk of acquiring HIV nationally at 1 in 99.^3^ It is projected that 1 in 6 men who have sex with men (MSM) will acquire HIV in their lifetime, which is over 70 times the lifetime risk of heterosexual men. The lifetime risk is highest in African American men who have sex with men (at nearly one in two) and lowest in in white men who have sex with men (one in eleven). In 2014, the CDC recommended the use of Truvada_®_ (tenofovir disoproxil fumarate and emtricitabine) as a method to prevent HIV transmission for three patient populations if they have a substantial risk of acquiring HIV: men who have sex with men, IV drug users, and heterosexually active couples.^4^

HIV specialists noted that patients are more likely to seek care from a primary care physician to start PrEP therapy; thus PrEP may be managed more appropriately by primary care physicians.^5^ We assessed if family physicians routinely screen for high risk sexual behaviors and if there are physician biases towards prescribing PrEP for MSM that prevent moving this preventative therapy into the family physician’s domain of treatment.

## Methods

The study protocol was approved by the Institutional Review Board at the University of Kansas School of Medicine-Wichita. A confidential email survey was sent to the 85 members of a practice-based research network of family physicians in Kansas, the Family Medicine Research and Data Information Office (known as FM RADIO). The survey was sent with two follow-up emails for non-responders. Surveys were sent via SurveyMonkey_®_ which provided a link to an online survey and allowed for an anonymous response.

## Results

The response rate was 20 (23.5%). Sixteen respondents identified their sex; eleven (69%) were males. The average age of the respondents was 55 years with a range of 31 to 74 years. Sixteen respondents revealed their practice county. Seven respondents practiced in a rural county (44%), six practiced in an urban county (38%), and three practiced in counties with a mid-sized regional community (19%).

Respondents revealed they were not familiar with the CDC Preexposure Prophylaxis (PrEP) for the Prevention of HIV practice guidelines^4^. Of 18 respondents, only one (6%) was extremely familiar and seven (39%) were not familiar at all. Two of eighteen respondents (11%) had prescribed the recommended PrEP therapy and HIV antiretroviral medication Truvada_®_.

Table 1 reveals the respondents’ practices in taking a sexual history. As shown, few always take a sexual history on new patients. Few always ask important sexual history questions. Table 2 reveals survey responses related to the willingness to prescribe PrEP in certain patient populations. Most family physician respondents were willing to prescribe PrEP to their patients. Table 3 reveals barriers in prescribing PrEP therapy.

Eleven of eighteen respondents (61%) agreed that PrEP belongs in the primary care domain of treatment. Only four (22%) agreed that PrEP belongs only in the HIV specialist’s domain. Eighty-two percent stated they would be willing to prescribe PrEP with more education and training. Ten of 17 respondents (59%) agreed that PrEP should be covered by private insurance.

Table 4 reveals the conditions when family physician respondents would be willing to prescribe PrEP. Most respondents (70%) would be willing with PrEP education and training.

## Discussion

This preliminary study indicated a need for increased family physician screening of new patients for high risk sexual behaviors who would be eligible for PrEP. In our sample, no apparent bias was noted against prescribing PrEP for men who have sex with men, as survey participants were equally willing to prescribe Truvada_®_ to MSM and heterosexual couples at high risk for acquiring HIV. Based on the study results, the limited knowledge of PrEP guidelines and their use in clinical practice are significant limiting factors to increasing prescribing practices in the family medicine community rather than a perceived ethical dilemma of prescribing Truvada_®_ to men who have sex with men. Yet, it is difficult to assess the “true” willingness for family physicians to prescribe a medication that a significant number are unfamiliar with. As a result, an increase in continuing medical education about Truvada_®_ could increase its prescribing in the family medicine community.

The strengths of the study include a mixture of both urban and rural participants in communities with varying populations. The small sample size may be due to the controversial nature of the topic which may have shifted the responses towards a more positive side if those who disagreed abstained from the survey, thereby, reflecting a potential sampling bias.

Even with only two study participants not willing to prescribe PrEP, this could be significant in rural areas as there are few primary care physicians available. There is an estimated 3,333 individuals living with HIV in Kansas as of December 2013, which is likely lower than other geographic regions in the United States and could limit physician exposure to HIV management and education on the topic. 6 Further, the lack of awareness of PrEP guidelines as shown by the survey responses may have decreased participation in a cohort known to respond to survey requests.

## Figures and Tables

**Table 1:** Selected responses to survey items related to sexual history practices (n, %).

Survey Item	Never	Rarely	Sometimes	Frequently	Always
I take a sexual history on all new patients (n = 19).	0 (0%)	4 (21%)	6 (32%)	6 (32%)	3 (16%)
When I take a sexual history, I ask about the patient’s use of safe sex practices (n = 18).	1 (6%)	6 (33%)	5 (28%)	5 (28%)	1 (6%)
When I take a sexual history, I ask if the patient has had multiple sexual partners in the last 6 months (n = 18).	1 (6%)	7 (39%)	5 (28%)	4 (22%)	1 (6%)
When I take a sexual history, I ask if the patient has sex with men, women, or both.	2 (11%)	7 (39%)	4 (22%)	3 (17%)	2 (11%)

**Table 2:** Respondents willingness to prescribe PrEP in certain patient populations (n, %).

I would be willing to prescribe Truvada_®_ for HIV Preexposure Prophylaxis (PrEP) to the following patient population(s) if there are no contraindications (n = 17):					
	Strongly Agree	Agree	Neutral	Disagree	Strongly Disagree
Sexually active adult men who have sex with men (MSM) at substantial risk of HIV acquisition.	8 (47%)	5 (29%)	2 (12%)	0 (0%)	2 (12%)
Heterosexually active men and women at substantial risk of HIV acquisition.	8 (47%)	5 (29%)	2 (12%)	0 (0%)	2 (12%)
Heterosexually active men and women whose partners are known to have HIV infections to protect the uninfected partner during conception and pregnancy.	10 (59%)	3 (18%)	3 (18%)	0 (0%)	1 (6%)

**Table 3:** Barriers to prescribing PrEP therapy (n, %).

Barrier	Strongly Agree	Agree	Neutral	Disagree	Strongly Disagree
Limited or no knowledge of PrEP guidelines (n = 17)	8 (47%)	4 (24%)	4 (24%)	1 (6%)	0 (0%)
Concerned about side effects of Truvada as a prophylactic medication (n = 17)	1 (6%)	3 (18%)	11 (65%)	2 (12%)	0 (0%)
PreP therapy could increase the likelihood of sexually transmitted infections among men who have sex with men (n = 17)	1 (6%)	1 (6%)	7 (41%)	6 (35%)	2 (12%)
There are patient adherence and compliance issues with PrEP that will decrease its efficacy (n = 17)	0 (0%)	5 (29%)	8 (47%)	3 (18%)	1 (6%)
Prescribing will increase high risk sexual behaviors among men who have sex with men (n = 17).	1 (6%)	1 (6%)	5 (29%)	7 (41%)	3 (18%)
PrEP therapy will decrease safe sex practices among men who have sex with men (n = 17)	1 (6%)	3 (18%)	4 (24%)	8 (47%)	1 (6%)
There would be a stigma or backlash in the office if I prescribe PrEP therapy (n = 17)	1 (6%)	0 (0%)	3 (18%)	11 (65%)	2 (12%)
Limited time or resources for patient education about PrEP therapy (n = 17)	1 (6%)	6 (35%)	8 (47%)	2 (12%)	0 (0%)
No desire to prescribe a medication that requires lab work and follow up every 3 months (n = 17)	0 (0%)	3 (18%)	9 (53%)	4 (24%)	1 (6%)
Perceived moral and/or ethical dilemma prescribing PrEP to men who have sex with men (n = 16)	0 (0%)	2 (13%)	3 (19%)	8 (50%)	3 (19%)

**Table 4:** Conditions when respondents would be willing to prescribe PrEP (n = 20).

Condition	n (%)
I received PrEP education and training.	14 (70%)
PrEP is covered by private insurance.	5 (25%)
I know other family physicians prescribe PrEP.	7 (35%)
I did not have to prescribe it to men who have sex with men.	1 (5%)
I read research that demonstrates its efficacy in HIV prevention.	7 (35%)
Under no circumstances would I prescribe PrEP therapy.	1 (5%)
